# A clinical investigation treating different types of fibroids identified by MRI-T2WI imaging with ultrasound guided high intensity focused ultrasound

**DOI:** 10.1038/s41598-017-11486-5

**Published:** 2017-09-07

**Authors:** Wen-Peng Zhao, Jing Zhang, Zhi-Yu Han, Jin-Peng Yao, Xiang Zhou, Ping Liang

**Affiliations:** 1 Department of Ultrasound, National Cancer Center; Cancer Hospital, Chinese Academy of Medical Sciences and Peking Union Medical College, No. 17 Panjiayuan Nanli, Chaoyang District, Beijing, 100021 China; 2 Department of Interventional Ultrasound, Chinese PLA General Hospital, No. 28 Fuxing Road, Haidian District, Beijing, 100853 China

## Abstract

Clinical data from 172 cases of uterine fibroids with different appearances on MRI-T2WI and accepted ultrasound guided high intensity focused ultrasound (USgHIFU) treatment were retrospectively analyzed. This study aimed to evaluate the clinical safety and efficacy of ablating different types of fibroids, classified by T2-weighted magnetic resonance imaging (MRI-T2WI). Based on MRI-T2WI signal intensities, uterine fibroids were classified as three types: hypointensive (52 cases), isointensive (64 cases) and hyperintensive (56 cases). Evaluation parameters including treatment time, ablation efficiency, percentage non-perfused volume, fibroid reduction rate, adverse reactions, symptom severity scores (SSS) and re-intervention rate were assessed from 3 months to 1 year. The percentage non-perfused volume and ablation efficiency of hyperintensive uterine fibroids were lower than those of isointensive and hypointensive uterine fibroids. All fibroids shrunk and the SSS continued to reduce at 3 and 6 months after treatment respectively. At 12-month postoperative assessments, hypointensive fibroids continued to shrink, while the isointensive fibroids enlarged but remained smaller than pre-treatment. The incident rate of postoperative Society of Interventional Radiology B-class (SIRB-class) adverse events showed no significant differences. The re-interventional rate of hyperintensive fibroids was higher than in isointensive and hypointensive groups. USgHIFU ablation of all types of fibroids were safe and effective.

## Introduction

Uterine fibroids are common benign neoplasms among reproductive aged women, impairing their quality of life^1^. Approximately 10–20% of women with uterine fibroids exhibit symptoms, such as menorrhagia, heavy bleeding, infertility, pain, frequent urination and constipation. Surgical interventions such as laparoscopic or hysteroscopic myomectomy or hysterectomy are the most common methods of treatment. However, the use of myomectomy is limited by location and size of the fibroid, and with complications such as uterine rupture^2^. Moreover, hysterectomy is not suitable for women who have strong desire for future pregnancy. Therefore, non-surgical treatment is highly required for some women with uterine fibroids.

There is a trend emerging to move surgical treatments from invasive to non-invasive. Consistent with this trend, high-intensity focused ultrasound (HIFU) draws clinicians’ attention and patients’ acceptance due to its non-invasive features and minimal complications. The principle of HIFU ablation relies on high energy absorption in the focal area of a tumor, which is based on the hyperacoustic tissue penetrability and focalization characteristics^3–5^. The thermal effect generated by ultrasound in the lesion can raise the temperature of the target area rapidly to between 60 °C and 100 °C within 0.5–1.0 seconds, causing coagulation necrosis in the target tissue and leaving the surrounding normal tissue intact. A large number of clinical studies have confirmed the safety and effectiveness of HIFU, which has been widely used in treatments for solid tumors such as uterine fibroids, prostate gland cancer, pancreatic cancer, liver cancer and bone tumor. The clinical research regarding HIFU ablation of uterine fibroids revealed that HIFU is a safe, cost-effective and non-invasive treatment with promising applications^4–7^. It is possible that HIFU could be a better treatment of choice, especially for patients desiring future fertility^8^.

The two guiding models that help HIFU to ablate lesions are magnetic resonance imaging (MRI) and ultrasound (US). While clear and accurate monitoring images and intraoperative temperature measurements can be obtained by MRI-guided HIFU (MRIgHIFU), it is expensive, requires complicated operations, and exhibits poor real-time performance. Conversely, US guided HIFU (USgHIFU) is relatively inexpensive, user-friendly, and exhibits good real-time performance. However, the images from USgHIFU are easily interfered by intestinal gas, scar, subcutaneous fat thickness *et al*. And intraoperative temperature cannot be measured during USgHIFU treatment, and instead is evaluated semi-quantitatively by the gray level.

Studies using MRIgHIFU to ablate uterine fibroids^6, 9, 10^ showed that the percentage non-perfused volume which is defined as the percentage rate of the non-perfused volume comparing with fibroid volume after HIFU treatment on enhanced MRI, immediately after ablation was approximately 10% to 50%, and rarely over 70%. One study^11^ has revealed that the pathologic structure of fibroids, which is associated with absorption of ultrasound energy in uterine fibroids, has a close relationship with percentage non-perfused volume, and that the decrease rate of fibroids 6-month post-operation ranged from 20% to 50%. Studies have shown that the percentage non-perfused volume and the decrease rate of MRI-T2WI hyperintensive fibroids are lower than those of isointensive and hypointensive fibroids^10^. This difference may lead to discrepancies in clinical effectiveness among patients with fibroids of different signals.

Another research using USgHIFU to ablate uterine fibroids has revealed that the percentage non-perfused volume immediately following ablation could reach 77.1% with differences in the percentage non-perfused volume of different MRI-T2WI signal fibroids^12^. However, there is very limited data regarding the long-term effectiveness and safety of USgHIFU ablation of uterine fibroids, as well as long-term effectiveness of USgHIFU ablation of uterine fibroids with different MRI-T2WI signals. Therefore, this retrospective study analyzed the safety and effectiveness of USgHIFU ablation of uterine fibroids with different MRI-T2WI signals.

## Materials and Methods

### Patient recruitment

**Figure 1:**
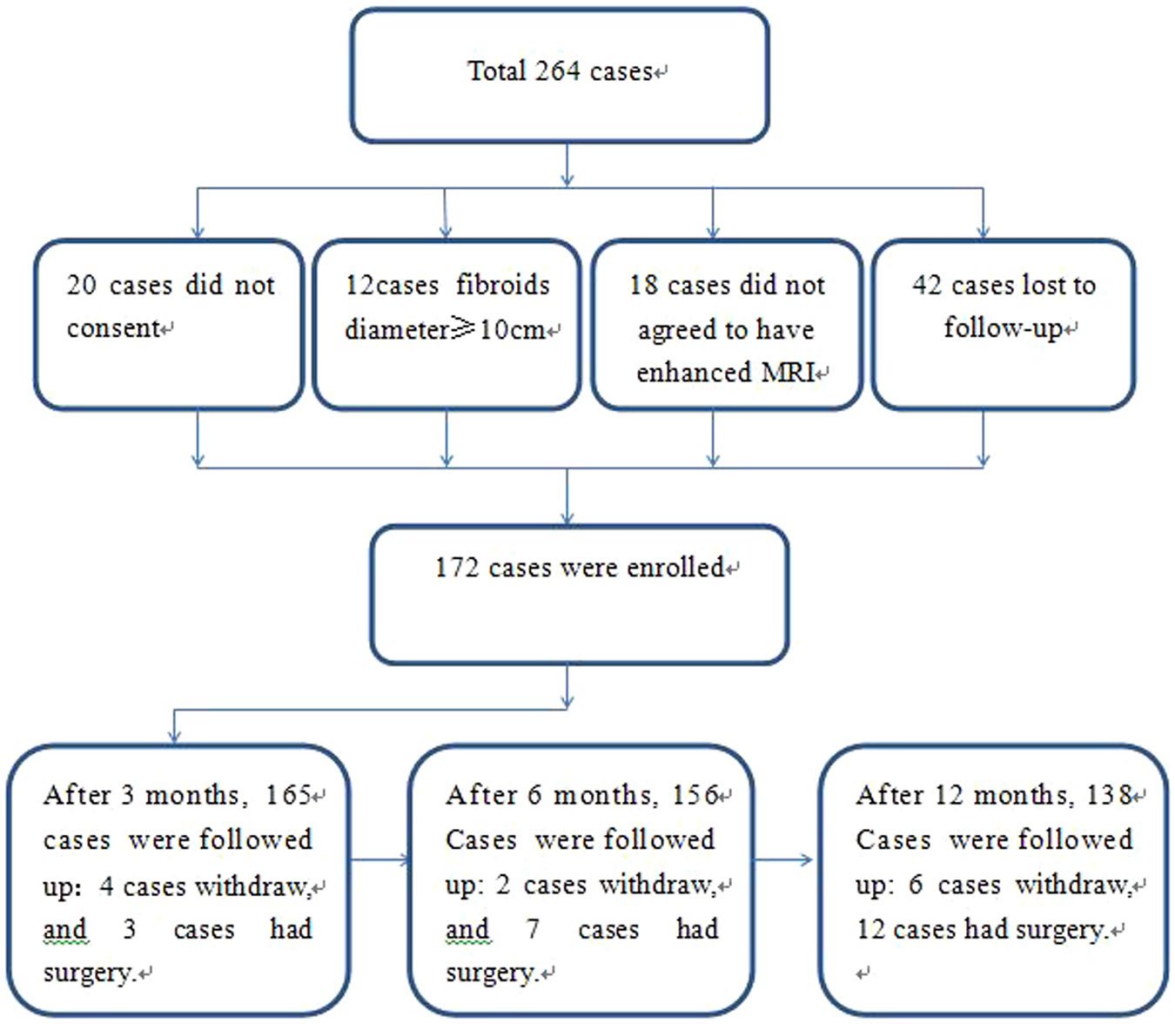
The flow of participants through the study.

264 cases of uterine fibroids received USgHIFU therapy between January 2012 and March 2015. Among them, 172 cases were in accordance with the inclusive criteria (please refer to the flow Fig. 1 for details), which completed 3 to 12 month follow-up visits, were included in this study. In this sample, the temporal collection of patients partly overlapped with our prior study (N = 42)^12^. All of the patients received plain and enhanced MRI scans before the operation. For patients with multiple fibroids, USgHIFU focused on the single dominant fibroid that caused symptoms, and did not treat the remaining minor fibroids. This study protocol was carried out in accordance with the principles of the Declaration of Helsinki and had obtained the ethics committee approval from from General Hospital of People’s Liberation Army. All patients signed an informed consent before operation.

### Inclusion and exclusion criteria

Patients fulfilling the following criteria were included in this study: (1) premenopausal women aged over 18 years old; (2) presence of symptoms such as prolonged menstruation, menometrorrhagia, frequent micturition, constipation, infertility, hypogastric pain and soreness of waist; (3) have no plan of conception in the next 6 months; (4) patients with imitative positioning preoperatively and secure acoustic passageway with visible fibroids under the monitoring ultrasound; (5) patients who can communicate fluently with medical staff; (6) patients who agreed to have plain and enhanced MRI scan before and after operation; (7) maximum diameter of fibroid between 3 and 10 cm (Table 1).

**Table 1:** Inclusion and exclusion criteria.

Item	Inclusion	Exclusion
Age(y)	≥18, ≤50	<18, >50
Related symptoms	Yes	No
Plan of conception in 6 Mon	No	Yes
Acoustic passageway under the monitoring ultrasound	Security	insecurity
Communicate fluently with medical staff	Yes	No
Enhanced MRI scan before and after operation	Yes	No
Maximum diameter of fibroid(cm)	3 cm ≤ D ≤ 10 cm	<3 cm, >10 cm
Pregnant or menstrual period	No	Yes
Suspected uterine malignant tumor	No	Yes
Pelvic inflammation disease or uncontrollable systemic disease	No	Yes
Lie in the prone position for 2 hours	Yes	No
Spontaneous necrosis of fibroids	No	Yes
Abdominal radiotherapy or severe connective tissue diseases	No	Yes

Patients with following conditions were excluded in this study: (1) women who were pregnant or during menstrual period; (2) patients who had contraindications of MRI exam or contrast agents containing gadolinium; (3) patients who were suspected to have a uterine malignant tumor; (4) patients with acute pelvic inflammation disease or uncontrollable systemic disease; (5) patients who could not lie in the prone position for 2 hours; (6) patients whose preoperative MRI exam showed obvious spontaneous necrosis of fibroids; (7) patients who had history of high dose abdominal radiotherapy or severe connective tissue diseases (Table 1).

### Equipment

The treatment was performed with a US-guided HIFU tumor therapeutic system, (JC, Chongqing Haifu (HIFU) Tech Co.,Ltd., Chong qing, China), and B-mode ultrasound (2.5–3.5-MHz, Esaote MyLab70, Italy) which was situated in the center of the high-intensity focused ultrasound transducer. Transducer of HIFU was 20 cm in diameter, 0.8 MHz in operating frequency, 0–400 W in the range of output power, 3000–20000 W/cm^2^ in the range of acoustic spatial-peak temporal-average intensity, 15 cm in focal length and 1.5 mm × 1.5 mm × 10 mm in the dimensions of focal region. The current platforms was used in Asia, Africa, Europe and Latin America; this had passed the European CE certification.

### Pre-operation evaluation

Patients included in this study all had preoperative plain and enhanced MRI examinations to assess the position and size of the uterus as well as the quantity, size, position and T2WI signal of fibroids. All MRI data were evaluated and measured by two experienced doctors. The volumes of the fibroids were measured on T2WI signals by three dimensions: length diameter (D1), anteroposterior diameter (D2) and transverse diameter (D3) in triplicate, and then an average value was obtained. The volumes according to the formula of ellipsoid were calculated as follows: V = 0.5233 × D1 × D2 × D3^13^.

**Figure 2:**
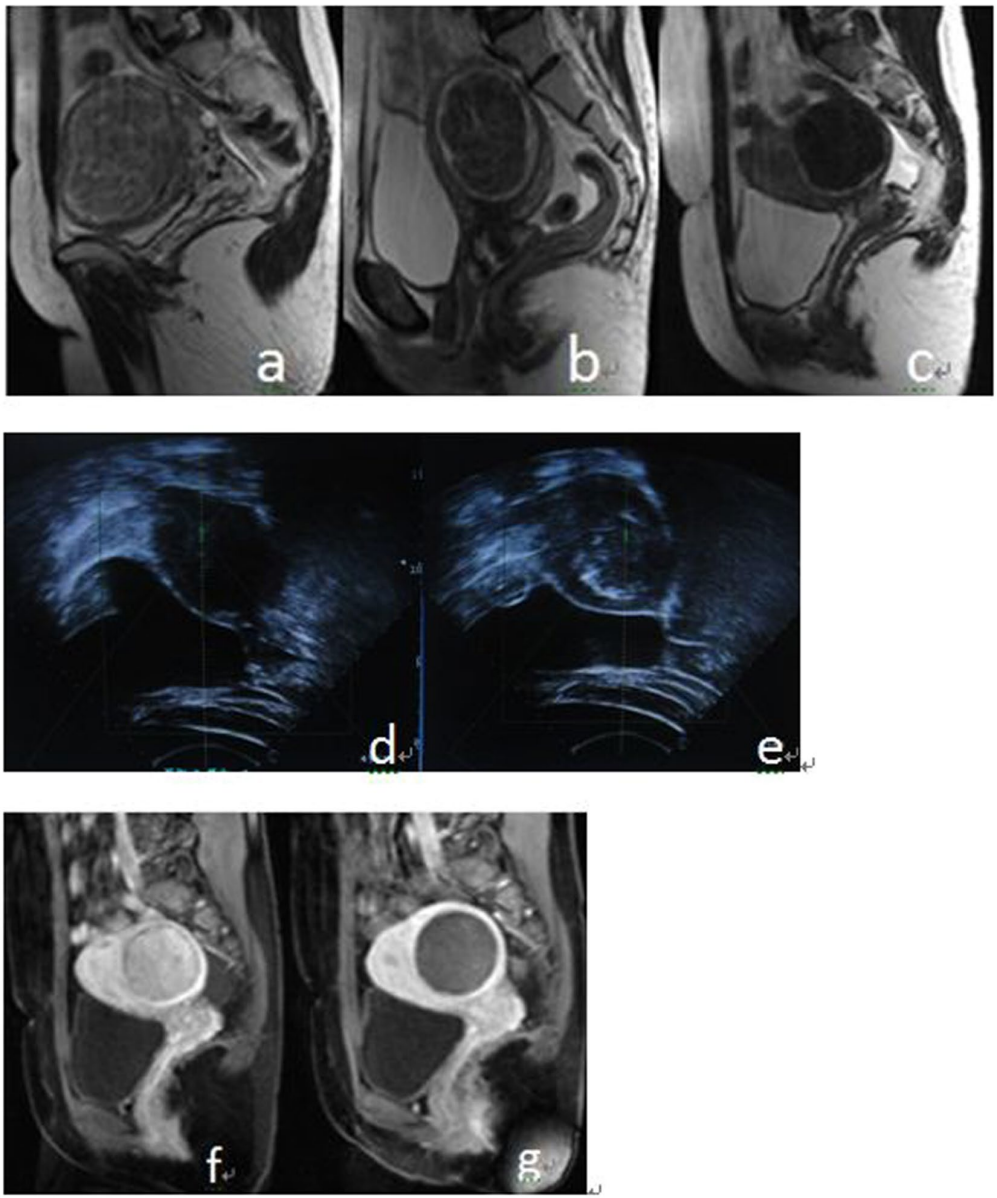
(**a**–**c**) MRI appearance of different types of uterine fibroids on T2-weighted sagittal MRI before treatment; (**a**) hypointense; (**b**) isointense; (**c**) hyperintense. (**d**–**e**) US images that show the ablation volume during the real-time USgHIFU; (**d**) ultrasound image shows a uterine fibroid with hypoecho before treatment; (**e**) gray scale changes was observed after treatment. (**f**–**g**) Enhanced sagittal MRI before and after treatment, the NPV is visible inside uterine fibroids; (**f**) before treatment, g after treatment.

**Figure 3:**
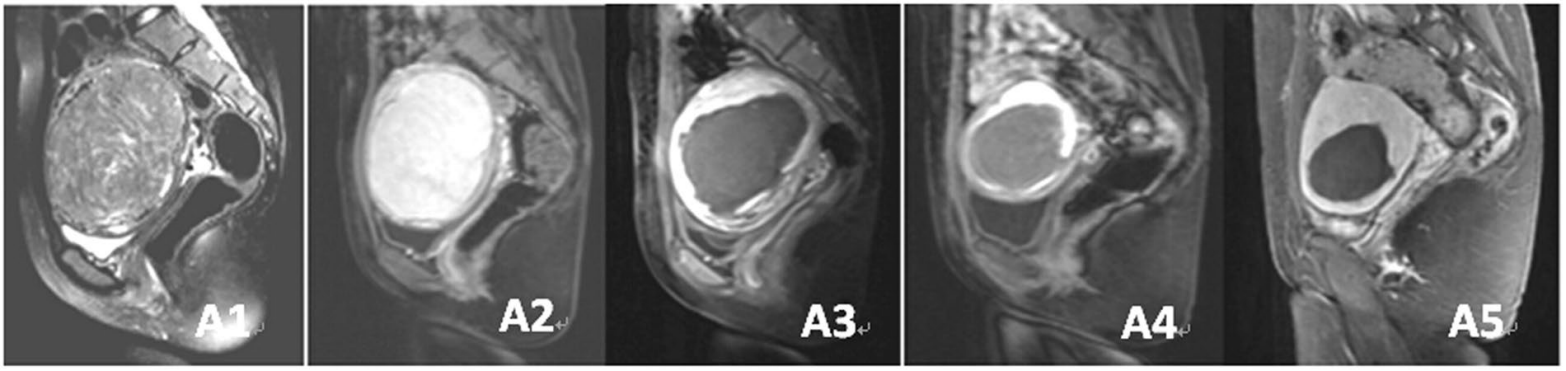
MRI of hyperintense fibroid before and after HIFU treatment: A1, T2WI-MRI before treatment; A2, fibroid shows enhancement on contrast-enhanced MRI before treatment; A3, 3 months after treatment; A4, 6 months after treatment; A5, 12 months after treatment.

Based on MRI-T2WI signal intensities, uterine fibroids were classified as three types^14^: (1) hyperintensive, signal intensity equal to or greater than the uterine muscle wall (Fig. 2a); (2) isointensive, signal intensity greater than skeletal muscle but lower than uterine muscle wall(Fig. 2b); (3) hypointensive, similar signal intensity to skeletal muscle (Fig. 2c).

### Process of USgHIFU

Three days prior to the operation, patients were required to have a semi-liquid to full liquid diet. An enema was provided within 12 hours pre-operation. Skin preparation, defatting and degassing were also required on the operation area. Sedation and analgesia were applied using fentanyl citrate (0.8–0.1 µg/kg) and midazolam (0.02–0.03 mg/kg) via intravenous injection, and repeated every 30 to 40 minutes. The sedation depth reached Ramsy Grade level 2 to 3 before operation. All procedures were performed by qualified doctors experienced in USgHIFU.

Patients lay in the prone position with the lower abdomen buried in a water bag full of degassed water. The lesion area was adjusted above the transducer. Fibroids could be accurately located by adjusting the directions and angles of the transducer, which was connected with monitoring to the ultrasonic probe. A vertical real-time ultrasound was used to observe the target area and adjacent structure. Treatment began from the dorsal of uterine fibroid to the ventral and the distances between the focus and the boundary of junctional and endometrium were at least 1 cm and 1.5 cm, respectively. This process was repeated on a section-by-section basis to achieve complete ablation of the planned, The thickness of one section was 5 mm. During the treatment, the operator could adjust the focal position, power and strength according to the patient’s reaction and the change of fibroid gray levels shown by real-time ultrasonic monitoring (Fig. 2d). The procedure was stopped when the lesion was almost covered by grayscale (Fig. 2e). Patients lay in the prone position for 2 more hours after the operation. Routine oral antibiotics were administrated postoperatively for 3 to 5 days and contraceptive measures were required for the next six months.

### Postoperative evaluations

Evaluation parameters including, operation time (min, from the beginning of ultrasonic irradiation to ending), ablation efficiency (mm^3^/s, ablated fibroid volume in 1 s sonication emission), percentage non-perfused volume (the percentage rate of the non-perfused fibroid volume comparing with whole fibroid after HIFU treatment on enhanced MRI, Fig. 2f,g), the decrease rate of fibroid, pain score (using the standard Visual Analogue Scale (VAS) Pain Score, and evaluated immediately after the operation), and adverse reactions. Patients also underwent plain and enhanced MRI scan immediately, 3 months, 6 months and 12 months after the operation, respectively, to evaluate postoperative ablation rate, decrease rate of fibroid, adverse reaction, SSS (symptom severity scores) and re-treatment rate (the proportion of patients requiring re-treatment after the first ablation). The occurrence of an adverse reaction was evaluated using the classification standard of Society of Interventional Radiology (SIR) complications.

### Statistical analysis

Normally distributed data were expressed as mean ± standard deviation, while non-normally distributed data were expressed as median and interquartile range. Comparisons among groups were made using multiple factors analysis of variance, *Mann*-*Whitney U* test, *t* test and chi-square test, with a *P* value < 0.05 to be considered as significant. Data were analyzed by SPSS 17.0software (SPSS, IBM, USA).

## Result

### Clinical characteristics of patients and fibroids

There were 172 patients who underwent treatment and completed the follow-up visits. The follow-up periods ranged from 3 to 12 months. The number of cases with hyperintensive, isointensive and hypointensive fibroids detected by MRI-T2WI were 56, 64 and 52, respectively. The average age of patients was 37.1 ± 6.0 (22–48) years old and the median fibroid volume was 60.4 (34.6–109.7) cm^3^. Among them, the median volume of hyperintensive fibroids in MRI-T2WI was 71.9 (42.6–191.2) cm^3^, which was significantly higher than fibroids in patients with hypointensive fibroid (*P* < 0.05, Tables 2 and 3).

**Table 2:** Baseline characteristics of all patients with uterine fibroids treated by USgHIFU.

Variable	Hyperintensive fibroid	Isointensive fibroid	Hypointensive fibroid	Total
Cases	56	64	52	172
Age (years)	35.1 ± 5.2	37.2 ± 6.4	38.7 ± 6.1	37.1 ± 6.0
BMI (kg/m^2^)	22.4 ± 2.5	21.6 ± 2.5	22.9 ± 2.9	21.9 ± 2.7
Fibroid location (AW/PW/LW)	21/11/24	26/17/21	22/12/18	69/40/63
Type of fibroid (IMF/SMF/SSF)	38/8/10	39/11/14	30/6/16	107/25/30
Median fibroid volume (cm^3^)	71.9 (42.6–191.2)	60.6 (30.7–101.3)	42.7 (32.8–73.6)	60.4 (34.6–109.7)
SSS (point)	34.5 ± 4.3	32.8 ± 6.0	31.1 ± 7.0	33.1 ± 6.9

BMI, body mass index; AW, anterior wall; PW, posterior wall; LW: lateral wall; IMF, intramural fibroid; SMF, submucous fibroid; SSF, subserous fibroid; SSS, symptom severity score.

**Table 3:** Baseline characteristics of three groups were compared.

Variable	Cases	Age	BMI	Fibroid locatin	Type of fibroid	fibroid volume	SSS
Hyper vs Iso (*P* value)	*P* = 0.72	*P* = 0.58	*P* = 0.86	*P* = 0.74	*P* = 0.70	*P* = 0.42	*P* = 0.48
Hyper***vs*** Hypo (*P* value)	*P* = 0.84	*P* = 0.86	*P* = 0.89	*P* = 0.76	*P* = 0.54	*P* = 0.01	*P* = 0.49
Iso ***vs*** Hypo (*P* value)	*P* = 0.56	*P* = 0.77	*P* = 0.90	*P* = 0.68	*P* = 0.36	*P* = 0.18	*P* = 0.58

VS, Versus; Hyper, Hyperintensive; Iso, Isointensive; Hypo, Hypointensive.

**Figure 4:**
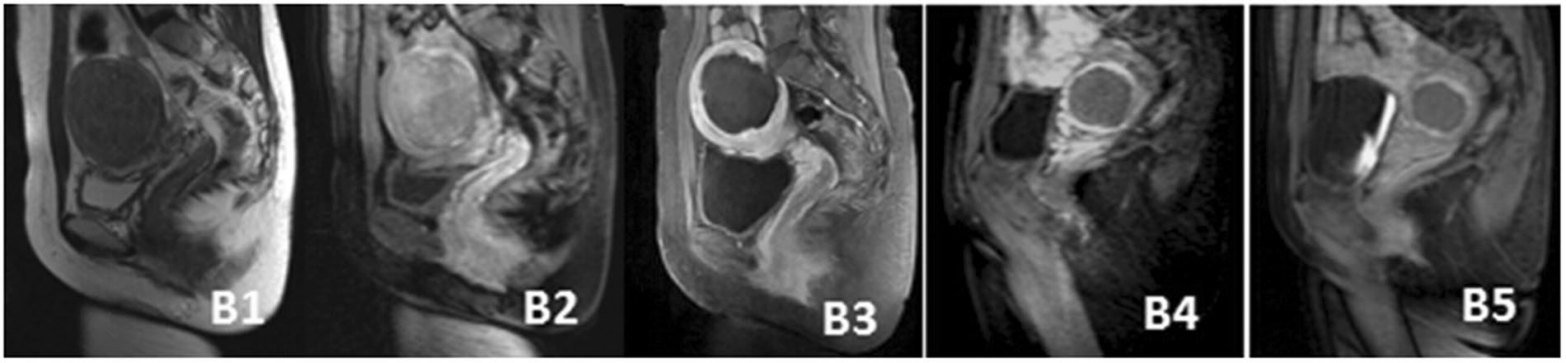
MRI of isointense fibroid before and after HIFU treatment: B1, T2WI-MRI before treatment; B2, fibroid shows enhancement on contrast-enhanced MRI before treatment; B3, 3 months after treatment; B4, 6 months after treatment; B5, 12 months after treatment.

**Figure 5:**
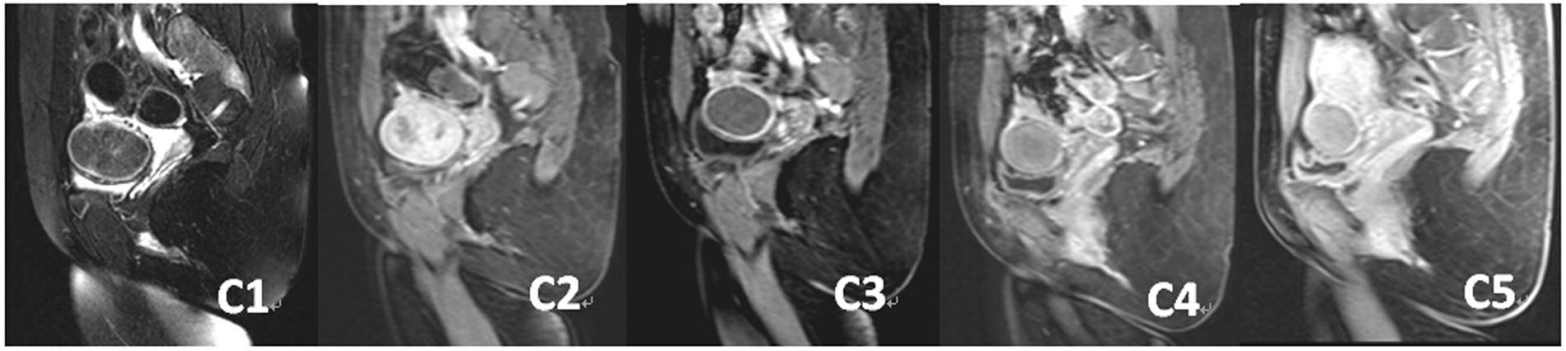
MRI of hypointense fibroid before and after HIFU treatment:C1, T2WI-MRI before treatment; C2, fibroid shows enhancement on contrast-enhanced MRI before treatment; C3, 3 months after treatment; C4, 6 months after treatment; C5, 12 months after treatment.

The average power of HIFU was 400 W and the median total treatment duration was 89 (62–154) min. The median treatment time duration for hyperintensive fibroids was 121 (76–181) min. The treatment duration for hypointensive fibroids was significantly shorter than for hyperintensive and isointensive fibroids (*P* < 0.05, Tables 2 and 3). The average percentage non-perfused volume was 76.2 ± 20.7% (12.5–100%), and the median ablation efficiency was 43.2 (22.5–81.6) mm^3^/s. The percentage non-perfused volume and the ablation efficiency of hyperintensive fibroid were significantly lower than isointensive and/or hypointensive fibroids (*P* < 0.05, Tables 4 and 5, Figs 3, 4 and 5).

**Table 4:** Treatment results of different types of uterine fibroids based on T2-weighted MRI treated by USgHIFU.

Variable	Hyperintensive fibroid	Isointensive fibroid	Hypointensive fibroid	Average
Treatment time (min)	121 (76–181)	90 (74–158)	79 (48–111)	89 (62–154.0)
Ablation efficiency (mm^3^/s)	33.1 (15.3–66.7)	38.2 (21.2–78.9)	49.0 (32.3–90.4)	43.2 (22.5–81.6)
Percentage non- perfused volume (%)	65.5 ± 27.8	75.4 ± 16.6	88.3 ± 15.1	76.2 ± 20.7

**Table 5:** Treatment results of three groups were compared.

Variable	Treatment time	Ablation efficiency	Percentage non- perfused volume
Hyper ***vs*** Iso (*P* value)	*P* = 0.17	*P* = 0.32	*P* = 0.10
Hyper ***vs*** Hypo (*P* value)	*P* = 0.006	*P* = 0.002	*P* = 0.008
Iso ***vs*** Hypo (*P* value)	*P* = 0.016	*P* = 0.002	*P* = 0.028

VS, Versus; Hyper, Hyperintensive; Iso, Isointensive; Hypo, Hypointensive.

### Follow-up results

A total of 165 cases were followed up at 3-month post-operation, the average volume reduction rate of fibroids was 38.1% (32.4–47.5%). There were no statistically significant differences between the volume reduction rate and the SSS level of hyperintensive, isointensive and hypointensive fibroids (*P* > 0.05, Tables 6 and 7). The total non-perfused volume of fibroid at 3-month postoperative was 62.3 ± 32.1% (0–100%) and the non-perfused volume of hyperintensive fibroid were significantly lower than isointensive and hypointensive fibroids (*P* < 0.05, Tables 8 and 9, Figs 3, 4 and 5).

**Table 6:** Fibroid volume reduction rate and descending level SSS of different types of uterine fibroids based on T2-weighted MRI at 3,6,12-month postoperative.

Variable	Hyperintensive fibroid	Isointensive fibroid	Hypointensive fibroid	Total
**3**-**month follow**-**up** (**n**)	53	61	51	165
Volume reduction rate (%)	37.9 (18.6–46.3)	40.1 (32.8–47.1)	39.7 (31.1–49.6)	38.1 (32.4–47.5)
Descending level of SSS(Point)	4.3 (2.2–7.9)	5.6 (3.4–9.1)	4.8 (3.5–8.7)	4.9 (2.6–9.2)
**6**-**month follow**-**up** (**n**)	49	58	49	156
Volume reduction (%)	53.2 (28.2–67.6)	59.4 (47.1–68.3)	45.7 (43.1–56.5)	50.1 (45.2–62.2)
Descending level of SSS(Point)	6.9 (1.2–11.2)	8.1 (3.4–12.6)	7.0 (3.2–11.4)	7.3 (3.1–15.6)
**12**-**month follow**-**up** (**n**)	40	52	46	138
Volume reduction (%)	35.4 (10.5–44.2)	50.2 (29.9–64.6)	61.3 (53.4–69.4)	50.9 (30.2–67.3)
Descending level of SSS(Point)	6.1 (0–12.6)	11.1 (2.1–13.8)	12.2 (4.2–14.1)	10.8 (2.8–13.4)

SSS, symptom severity score.

**Table 7:** Fibroid volume reduction rate and descending level SSS of three groups were compared at 3,6,12-month postoperative.

Variable	Hyper *vs* Iso −3 Mon	Hyper *vs* Hypo −3 M	Iso *vs* Hypo −3 M	Hyper *vs* Iso −6 M	Hyper *vs* Hypo −6 M	Iso *vs* Hypo −6 M	Hyper *vs* Iso −12 M	Hyper *vs* Hypo −12 M	Iso *vs* Hypo −12 M
Volume reduction rate (*P* value)	*P* = 0.56	*P* = 0.77	*P* = 0.89	*P* = 0.12	*P* = 0.049	*P* = 0.006	*P* = 0.48	*P* = 0.012	*P* = 0.045
Descending level of SSS (*P* value)	*P* = 0.32	*P* = 0.68	*P* = 0.43	*P* = 0.48	*P* = 0.84	*P* = 0.45	*P* = 0.01	*P* = 0.008	*P* = 0.57

VS, Versus; Hyper, Hyperintensive; Iso, Isointensive; Hypo, Hypointensive;

SSS, symptom severity score.

**Table 8:** Non-perfused volume (NPV) of different types of uterine fibroids based on T2-weighted MRI at 3,6,12-month postoperative.

Variable	Hyperintensive fibroid	Isointensive fibroid	Hypointensive fibroid	Total
3-month (%)	40.0 ± 31.0	66.7 ± 28.1	80.9 ± 17.4	62.3 ± 32.1
6-month (%)	34.6 ± 30.1	59.4 ± 32.2	76.8 ± 25.7	55.1 ± 35.1
12-month (%)	33.7 ± 28.5	21.5 ± 18.1	57.4 ± 30.2	43.7 ± 33.1

NPV, non-perfused volume.

**Table 9:** Non-perfused volume (NPV) of three groups were compared at 3,6,12-month postoperative.

Variable	Percentage non- perfused volume-3M	Percentage non- perfused volume-6M	Percentage non- perfused volume-12M
Hyper *vs* Iso (*P* value)	*P* = 0.01	*P* = 0.04	*P* = 0.04
Hyper *vs* Hypo (*P* value)	*P* = 0.00	*P* = 0.00	*P* = 0.46
Iso *vs* Hypo (*P* value)	*P* = 0.28	*P* = 0.23	*P* = 0.03

VS, Versus; Hyper, Hyperintensive; Iso, Isointensive; Hypo, Hypointensive;

NPV, non-perfused volume.

At the 6-month follow-up, the median volume reduction of fibroids and SSS of 156 patients were 50.1% (45.2–62.2%) and 7.3 points. The volume reduction rate of hypointensive fibroids was significantly lower than that in the hyperintensive and isointensive groups (*P* < 0.05). No statistically significant differences were observed in SSS between groups (Tables 6 and 7). The average non-perfused volume of fibroid at 6 months after operation was 55.1 ± 35.1% (0.0–100%). The non-perfused volume of hyperintensive fibroids was significantly lower than in isointensive and hypointensive fibroids (*P* < 0.05, Tables 8 and 9, Figs 3, 4 and 5).

By 12-month post-operation, a total of 138 cases were followed up, the median fibroid volume reduction rate and SSS descending levels were 50.9% (30.2–67.7%) and 10.8 points. The volume reduction rate of hypointensive fibroids was significantly higher than in the hyperintensive and isointensive groups (*P* < 0.05). The SSS descending level of hyperintensive fibroids was lower than that of isointensive and hypointensive groups (Tables 6 and 7). Non-perfused volume of hyperintensive and isointensive fibroids were significantly lower than that of hypointensive fibroids (*P* < 0.05, Tables 8 and 9, Figs 3, 4 and 5).

### Re-intervention

Twenty-two patients received re-intervention. Eighteen chose HIFU again, and 4 cases chose surgery. Among them, one patient with hypointensive fibroid chose surgery 6-month after treatment because of personal requirement even though her ablation results were satisfactory. By following up to 12-month postoperatively, a total of 12 patients in the hyperintensive fibroid group chose HIFU for re-intervention, which was much higher than patients with iso- and hypointensive fibroids (23.1% vs. 7.8%, *P* = 0.02; 23.1% vs. 5.4%, *P* = 0.038). More patients (54.5%) chose to have re-intervention at 12-month postoperatively than at 3- or 6-month postoperatively (Tables 10 and 11).

**Table 10:** Re-interventional rate of different types of uterine fibroids based on T2-weighted MRI at 3,6,12 months postoperative.

Variable	Postoperative −3 M	Postoperative −6 M	Postoperative −12 M	Total	Re-interventional rate (%)
Hyperintensive (n)	3	4	5	12	23.1
Isointensive (n)	0	1	4	5	7.8
Hypointensive (n)	0	1	2	3	5.4
Total (n)	3	7	12	22	12.8

**Table 11:** Re-interventional rate of three groups were compared at 3,6,12 months postoperative.

Variable	Hyper *vs* Iso	Hyper *vs* Hypo	Iso *vs* Hypo
Re-interventional rate (*P* value)	*P* = 0.02	*P* = 0.038	*P* = 0.34

VS, Versus; Hyper, Hyperintensive; Iso, Isointensive; Hypo, Hypointensive.

### Safety

During the treatment, the adverse events mainly included hypogastrium pain, coccydynia, skin burn, buttock pain, and lower limb radiation pain. The total incidence of pain was 82%. Patients with hyperintensive and isointensive fibroids reported higher but no statistical difference in incidences of pain than the hypointensive group (*P* > 0.05). The total pain score was 3.0 ± 0.7 (2–5) points and all the patients had score less than 5. The pain score of patients with hyperintensive fibroids was significantly higher than in the isointensive and hypointensive groups (*P* < 0.05, Tables 12 and 13). According to the classification standard of adverse event of international society for interventional radiotherapy (SIR), the most reported SIR-A adverse events during follow-up visits were vaginal discharge, pain at treatment area, coccydynia, and reddening of skin in the treatment area; no special management of adverse effects was needed. There were 10 cases of SIR-B degree adverse events. Two cases of hyperintensive fibroids showed skin bubbles, and required frequent local dressing changes for one week before recovery. One case of hyperintensive and one of isointensive fibroids exhibited postoperative pain in the sacrococcygeal region, which relieved after oral non-steroidal anti-inflammatory analgesic administration for 3 days. Two cases of hyperintensive, 1 case of isointensive and 2 cases of hypointensive fibroids exhibited numbness on one of the lower limbs between day 1–3 after operation. The pain eased after taking oral non-steroidal anti-inflammatory analgesics, vitamins and a neurotrophic drug for 7–30 days. One case with an isointensive fibroid showed perineum swelling and aching, and recovered after locally administered cold compress and analgesics for one week. The incident of SIR-B degree adverse events in patients with hyperintensive fibroids was 9.6%, which was higher (but not significantly) than in the isointensive and hypointensive groups (*P* > 0.05, Tables 12 and 13).

**Table 12:** The adverse events of patients with different types of uterine fibroids based on T2-weighted MRI during and after treatment.

Variable	Hyperintensive fibroid	Isointensive fibroid	Hypointensive fibroid	Total
Incidence of pain (%)	94.2 (49)	82.8 (53)	69.6 (39)	82.0 (141)
SSS	3.3 ± 0.8	3.0 ± 0.7	2.4 ± 0.5	3.0 ± 0.7
Incidence of SIR-B degree adverse event (%)	9.6 (5)	4.7 (3)	3.6 (2)	5.8 (10)

SSS, symptom severity score; SIR-B, Society of Interventional Radiology B-class.

**Table 13:** The adverse events of three groups were compared during and after treatment.

Variable	Incidence of pain	SSS	Incidence of SIR-B degree adverse event
Hyper *vs* Iso (*P* value)	*P* = 0.58	*P* = 0.39	*P* = 0.21
Hyper *vs* Hypo (*P* value)	*P* = 0.34	*P* = 0.000	*P* = 0.14
Iso *vs* Hypo (*P* value)	*P* = 0.69	*P* = 0.001	*P* = 0.72

SSS, symptom severity score; SIR-B, Society of Interventional Radiology B-class; VS, Versus; Hyper, Hyperintensive; Iso, Isointensive; Hypo, Hypointensive.

## Discussion

The clinical efficacy and safety of USgHIFU and MRI guided HIFU ablation on uterine fibroids have been fully investigated in prospective studies. This retrospective study may help to make an indirect comparison between the safety and efficacy of USgHIFU and MRI guided HIFU ablation of uterine fibroids.

In this study, the average volume reduction rate of fibroids using USgHIFU was higher than reports from other studies. Stewart^9^ showed that only 3% of the patients achieved more than 70% of volume reduction rate using MRI guidance. Funaki^15–17^ reported that the average volume reduction rate of fibroids was 49.0–60% with the help of MRIgHIFU. The results above are considerably lower than those found in our study. Moreover, those studies reported treatment time ranging from 170 to 210 min, which was longer than in our study. Our previous studies have confirmed that^18^ the features of MRI-T2WI hyperintensive uterine fibroids, including abundant myocytes, low tissue density, and high moisture content, prevent ultrasonic energy transformation into heat energy in the lesions, thus resulting in poor efficacy regarding fibroid volume reduction rate. In this study, the volume reduction rate and ablation efficiency of MRI-T2WI hyperintensive fibroids were lower than in the isointensive and hypointensive groups using USgHIFU, which was in accordance with previous reports^15^. The volume reduction rate of hyperintensive fibroids reached 63.2% in our study using USgHIFU, which was higher than the average volume reduction rate (28.5–36.2%) in another study using MRIgHIFU^10, 15^. Therefore, USgHIFU may be superior to MRI guided HIFU regarding fibroid volume reduction rate. This may be due to the device and clinical protocol of USgHIFU. Firstly, the core component of the HIFU ablation device is the transducer. USgHIFU is equipped with a spherical self-focusing transducer, which is user-friendly and exhibited a stable focal shape. By contrast, most of the MRI guided HIFUs are equipped with a phased array focusing transducer, which is difficult to operate, exhibits massive nonlinear effects and poorly forms focus beams in deep tissue area^19, 20^. Secondly, the protocols in other studies suggested a treatment area generally not exceed 50% of the fibroid volume. The transducer power during treatment is less than 200 W, which requires a strict treatment time and fibroid boundary. These clinical practices may lead to limited treatment scope and energy^21^.

The uterine fibroid percentage non-perfused volume was highly related to the reduction rate of fibroid during follow-up visits, and the reduction rate was a critical factor of remission of symptoms^9, 16^. Since the postoperative ablation rate in this study was significantly higher than that of MRI guided HIFU in other studies, the fibroid volume reduction rate and descending level of SSS in the follow-up visits were more obvious than in other studies. Studies of MRI guided HIFU in uterine fibroids ablation revealed^11, 15, 16^ that the average uterine fibroid volume reduction rate at postoperative 6-month and 12-month were 10.7–26.8% and 12.8–40.9%, respectively, which were both lower than the results of this study. In this study, no perfusion rate at postoperative 12-month follow-up remained at a high level, which indicated the possibility of continual shrinking in some fibroid lesions. Some researchers^15^ reported that the average reduction rate of hyperintensive fibroids using MRIgHIFU at the postoperative 6-month follow-up was −14.4 ± 42.1%; however, some fibroids began to regrowth afterwards. At 12-month postoperation, some fibroid volume enlarged. Compared with isointensive and hypointensive fibroids, the postoperative reduction of hyperintensive fibroids was much lower, which may be because the latter has more abundant cells and higher proliferation rate. In this study using USgHIFU, no significant difference in volume reduction rate at postoperative 3-month follow-up was observed among the 3 types of fibroids (*P* > 0.05). By the postoperative 6-month follow-up, the volume reduction rate of hyperintensive fibroids was similar to isointensive fibroids, and significantly higher than hypointensive fibroids (*P* < 0.05). At the postoperative 12 months, the volume reduction rate of hyperintensive fibroids was significantly lower than the hypointensive group (*P* < 0.05) and also showed a lower trend than the isointensive group (*P* > 0.05). These results indicated that although the volume reduction rate of hyperintensive fibroids was not so satisfactory, the fibroids quickly shrunk within 3-month postoperatively and reached their peak 6 months post operation. Because some fibroids may start to regrowth by 12 months post operation, the volume reduction rate dropped significantly by then. The volume reduction rate and SSS reduction level of isointensive fibroids increased consistently from 3 to 6-month post operation. However, similar to hyperintensive fibroids, at 12-month post operation, some isointensive myoma may regrowth. Unlike hyper- and isointensive fibroids, hypointensive fibroids showed a consistent volume and SSS reduction from 3 to 12 months post-operation. Since the hypointensive no perfusion rate was still high at 12 months postoperation, there was high possibility of continual fibroid volume shrinking. Therefore, a great difference in fibroid volume reduction rate can be observed in this study as well as others. Although the fibroid volume reduction rate in hyperintensive fibroids was lower than in the isointensive and hypointensive groups, some cases received obvious diminishment of fibroid volume. In future studies, if we can perform further subgroup analysis of hyperintensive fibroids, we may distinguish certain types of fibroid suitable for HIFU ablation.

To a certain extent, re-intervention can indirectly reflect the efficacy of clinical procedure. Funakai^9^ showed the re-interventional rate of hyperintensive fibroids was higher than that of isointensive fibroids at postoperative 6-month and 12-month follow-ups (9.1% vs. 1.4% at postoperative 6-month, and 21.6% vs. 2.9% at postoperative 12-month). In this study, the re-interventional rate of hyperintensive fibroids was higher than in the previous study. Given that the re-intervention rate was negatively related with percentage non-perfused volume, the percentage non-perfused volume of fibroid this study was higher than the previous study^9^. Therefore, improved fibroid ablation rates can greatly reduce the risk of re-intervention.

Regarding the safety of this procedure, the probability and severity of intraoperative and postoperative adverse events of hyperintensive fibroids were higher than in the isointensive and hypointensive groups. A high incidence of SIR-B degree adverse events in hyperintensive fibroids, especially the neural dysfunction, might be related to the longer ablation time and higher ultrasonic energy^22^. Therefore, except for careful preoperative examinations to help screen out the most difficult subtype of ultrasonic ablation, clinicians are required to avoid long-term intervention and high ultrasonic energy. Similar to other studies, no SIR-C degree and higher degree adverse events were observed. These results indicated that the treatment of uterine fibroids by USgHIFU was safe and efficient.

Even though this study provides evidence of promising use of USgHIFU, some shortcomings of this study may limit the wide application of USgHIFU. This non-randomized controlled study is a retrospective study with a small sample size. Moreover, redistribution of hormonal receptors, proliferation of myocytes and reestablishment of fibroid vascular network after ultrasonic ablation are still not clear, and worth more in-depth evaluation.

## Conclusion

To summarize, USgHIFU was safe and effective for patients of uterine fibroids with different appearances on MRI-T2WI. The re-interventional rate of hyperintensive fibroids was higher than in isointensive and hypointensive groups. In comparison to the published data in literature review^9–11, 15–17^, clinical outcomes and efficacy of USgHIFU in uterine fibroid ablation treatment seemed to be improved comparing with MRI guided HIFU ablation.
